# Design and evaluation of a multi-epitope assembly Peptide (MEAP) against herpes simplex virus type 2 infection in BALB/c mice

**DOI:** 10.1186/1743-422X-8-232

**Published:** 2011-05-16

**Authors:** Xingsheng Wang, Guangyan Xie, Jianming Liao, Dengke Yin, Wenyan Guan, Mingjie Pan, Jingnian Li, Yuexi Li

**Affiliations:** 1 Huadong Research Institute for Medicine and Biotechniques, Nanjing, 210002, China; 2 School of Life Science and Technology, China Pharmaceutical University, Nanjing, China, 210009; 3 Department of Pharmacy, Anhui University of Traditional Chinese Medicine, Hefei, China, 230038; 4 Department of Immunology and Microbiology, Anhui Agriculture University, Hefei, China, 230038

## Abstract

**Background:**

Human herpes simplex virus (HSV) 1 and 2 causes oral, ocular, or genital infections, which remains a significant health problem worldwide. HSV-1 and -2 infections in humans range from localized skin infections of the oral, ocular, and genital regions to severe and often disseminated infections in immunocompromised hosts. Epitope based vaccination is a promising mean to achieve protective immunity and to avoid infections with Human herpes simplex virus type 2 (HSV-2).

**Methods:**

The twelve selected epitopes, six B cell epitopes from different glycoprotein of HSV-2 (amino acid residues 466-473 (EQDRKPRN) from envelope glycoprotein B, 216-223 (GRTDRPSA) from C, 6-18 (DPSLKMADPNRFR) from D, 483-491 (DPPERPDSP) from E, 572-579 (EPPDDDDS) from G and 286-295 (CRRRYRRPRG) from I glycoprotein of HSV-2), four CD4^+ ^T cell epitopes (amino acid residues 21-28 (NLPVLDQL) from D, 162-177 (KDVTVSQVWFGHRYSQ) from B, 205-224 (KAYQQGVTVDSIGMLPRFIP) from D and 245-259 (KPPYTSTLLPPELSD) from D) and two CD8^+ ^T cell epitopes (amino acid residues 10-20 (KMADPNRFRGK) from D and 268-276 (ALLEDPAGT) from D), are responsible for the elicitation of the neutralizing antibodies and cytotoxic T lymphocytes (CTLs) that impart protective immunity to the host. In this study, all above epitopes were inserted into the extracellular fragment (amino acid residues 1-290) of HSV-2 glycoprotein D to construct multi-epitope assembly peptides (MEAPs) by replacing some non-epitope amino acid sequences. The epitope independency of the MEAPs was predicted by three-dimensional software algorithms. The gene of the selected MEAP was expressed in *E.coli *BL21(DE3), and its protective efficacy against HSV-2 infection was assessed in BALB/c mice.

**Results:**

The MEAP, with each inserted epitopes independently displayed on the molecule surface, was selected as candidate proteins. The results showed that the MEAP was highly immunogenic and could elicit high titer neutralizing antibodies and cell-mediated immune responses.

**Conclusions:**

The MEAP provided complete protection against infection with HSV-2 in mice, which indicates that it might be a potential candidate vaccine against HSV-2.

## Background

Human herpes simplex virus (HSV) 1 and 2 cause oral, ocular, and genital infections, which construct a significant health problem worldwide. HSV-1 and -2 infections in humans range from localized skin infections of the oral, ocular, and genital regions to severe and often disseminated infections in immunocompromised hosts [1]. After primary infection of mucosal epithelial cells, the virus establishes lifelong latency in sensory neurons, from which it periodically reactivates [2]. After reactivation, the virus migrates along the axons and infects cells to the site of primary infection, causing painful blisters on the surface of the lips in the case of HSV-1, or of the genital mucosa for the closely related HSV-2 [3].

Four glycoproteins of the HSV-2, glycoprotein B (gB), gD, gH and gL, have essential functions for HSV-2 entering into the host cells [4]. The cooperation of gB, the heterodimer gH/gL, as well as gD and the gD receptor are required in entering the plasma or endosomal membrane of host cells [5,6]. The function of gD in viral infectivity has been associated with the adsorption-penetration process. It binds to the host cell at the positions of 52, 60 and 197-199 of gD in the amino acid sequence. GB and gL, with the help from gK, are also importantly associated with the adsorption-penetration process [7,8].

During the last decade, HSV vaccine development has primarily focused on various forms of recombinant glycoprotein. Recently, many approaches in vaccine development have appeared, including one chemically synthesized peptides covering only a small region of the amino acid sequence of a protein [9]. It was reported that B cell epitopes from the amino acid sequence of gD2 could induce mice to produce antibodies against a potent and type-common antiviral activity, and some B cell epitopes of HSV-2 glycoprotein have been identified [10].

Neutralization antibodies to HSV-2 (B cellular immunity) play a prominent role in prophylactic protection from infection in animal models, while CD4^+ ^T cell-based cellular immunity to HSV-2 may play an important role in controlling recurrent human disease. Despite previous emphasis on antibody (Ab) and CD8^+ ^T cell responses, there is growing evidence to support a pivotal role for the CD4^+ ^T cells in antiherpesvirus immunity. CD4^+ ^T cells are required for the protection of mice from HSV-2 challenge [11]. Severe herpetic infections are often seen in immunocompromised individuals with impaired CD4^+ ^T cell immunity, such as those with AIDS and transplant patients, which indicate that CD4^+ ^T cells are very important for protection against virus infection. It is believed that CD4^+ ^T cell responses are important for protection against HSV-2 infection. These findings, along with the important role of CD4^+ ^T, CD8^+ ^T and B cells, suggested that a successful immunoprophylactic and immunotherapeutic strategy against HSV-2 should include immunodominant CD4^+ ^T and CD8^+ ^T cell epitopes.

It is necessary to develop an effective vaccine against HSV-2 that can stimulate the body to produce both cell-mediated and humoral immunity. Therefore, it is expected that a novel HSV-2 vaccine should be developed through the selection of immunodominant epitopes of CD4^+ ^T, CD8^+ ^T and B cell. In this study, twelve selected epitopes, six B cell epitopes, four CD4^+ ^T and two CD8^+ ^T cell epitopes, were all inserted into the extracellular fragment (1-290) of HSV-2 glycoprotein D to construct multi-epitope assembly peptides (MEAPs) by replacing some non-epitope amino acid sequences. The results showed that the MEAP expressed by bioengineering had strong and specific antigenicity and was able to strongly induce Th1 and Th2 cytokine (IL-2, IL-4 and IFN-γ) secretion, providing a basis for the development of novel vaccines against HSV-2.

## Materials and methods

### Virus and cell lines

Vero cells were grown in medium 199 supplemented with 10% fetal calf serums 100 U/ml penicillin, 0. 1 g/L streptomycin (all from GIBCO, USA) for use. Target cells (NIH 3T3 cells) was kindly provided by Prof. Liu (Anhui Medical University, China) and used as target cells for CTL assay. Monolayers were infected with HSV-2, Sav strain. Inactivated virus was prepared by infection of cells at a multiplicity of 0.1. After 4 days, extracellular virus was isolated and heat-inactivated for 30 min at 56°C, and stored at -80°C. Infectious virus (for animal protection experiments) was prepared by infecting cells at a multiplicity of 10. Medium was removed at 18 h after infection, and the cells were scraped into a small volume. Cells were sonicated and debris was removed by low speed centrifugation. The supernatant was stored at -80°C. The number of infectious virus particles was determined by a plaque assay. Briefly, virus dilutions were plated on monolayers of Vero cells in six well trays. Overlay medium containing 0.5% methyl cellulose was added. After 3 days, the monolayers were fixed and subsequently stained with Giemsa. Plaques were counted and the titers expresses as pfu per ml.

### Design of MEAPs

The twelve selected epitopes, six B cell epitopes (gB2_466-473_, gC2_216-223_, gD2_6-18_, gE2_483-491_, gG2_572-579 _and gI2_286-295_), four CD4^+ ^T cell epitopes (gD2_21-28_, gD2_205-224_, gD2_245-259 _and gB2_162-177_) and two CD8^+ ^T cell epitopes (gD2_10-20 _and gD2_268-276_), which all identified by further experiments (data no shown), were inserted into the extracellular fragment (1-290) of HSV-2 glycoprotein D to construct multi-epitope assembly peptides (MEAPs) by replacing some non-epitope amino acid sequences [9,12]. In order to find a homology model for suitable protein structure template, homologous protein sequences were searched using the protein blast method in the PDB database of the United States National Center for Biotechnology Information (NCBI). The results showed that the highest score from 1JMA was about 84% (30% protein homology is considered as a condition of homology modeling). So, 1JMA was used as a template for homology modeling of the multi-epitope assembly peptides (MEAPs). The structures the MEAPs were analyzed by Accelrys Discovery Studio and Moe2008 software, only the MEAPs with inserted epitopes independently displayed on the surface were selected as candidate proteins.

### Molecular cloning, expression, and purification of MEAP

The gene of a selected MEAP was chemically synthesized (by Jinsite Company, China), and cloned into the sites between *Bam*HIand *Xho*Iof plasmid pET28a (+)-sumo to construct a recombinant expression vector of pET28a (+)-sumo-MEAP, and thereafter transformed into *E. coli *BL21 for expressing a sumo-MEAP fusion protein. The engineered *E.coli *BL21 was cultured and induced by 1mM IPTG (Promega) at 25°C for 4 h. The induced engineered *E. coli *BL21 was collected by centrifugation, and lysised by ultrasound, the lysis supernatant was harvested by centrifugation, and concentrated by ultrafiltration.

Ni Sepharose 6 Fast Flow (GE Healthcare, Sweden) was suspended by gently rocking the bottle, an adequate amount of slurry was draw into a container through a pipe. The gel was washed with cold PBS (4°C), and thereafter, mixed with the lysis supernatant containing the expressed sumo-MEAP fusion protein. After oscillation for 30 min and centrifugation at 500 rpm for 5 min at room temperature, the supernatant was discarded and the precipitated gel was resuspended with washing buffer and loaded to a column, the gel was washed completely with washing buffer. The proteins were sequentially eluted from the column by gradient imidazole solutions (25, 50, 100, 150, 200 and 250 mM) in Tris-HCl (250 mM pH 8.5). The eluted parts were collected for detection of the purified sumo-MEAP fusion protein by SDS-PAGE. The elution fusion protein was dialyzed against Tris-HCl (50 mM pH 8.0), then 50 μg of sumo-MEAP was digested with 1 μl of sumo proteinase (Biotsith, Suzhou, China) with 2 U/μl at 22°C for 16 h. The digestion mixture was loaded on a Ni Sepharose 6 Fast Flow (GE Healthcare, Sweden) column in order to eliminate the sumo and sumo-MEAP that was not digested by sumo proteinase, and then was detected by SDS-PAGE electrophoresis.

### Immunization of mice

Thirty KunMing Mice (4-6 weeks old, male), were randomly divided into three groups. Each group was subcutaneously injected with MEAP, inactivated vaccine and PBS respectively. The immunization program as follows, the first injection with 4 μg of the purified MEAP (98%) emulsified in complete Freund's adjuvant, after 2 weeks, 2 μg of the purified MEAP emulsified in incomplete Freund's adjuvant was injected subcutaneously. The third injection was as that of the second. PBS, or inactivated vaccine was injected using the same procedure as MEAP immunization. Serum samples were collected on weeks 2, 3, 4 and 5 weeks after the first injection. Serum samples were separated and stored at -20°C for further assays. Two weeks after the final immunization, the mice were sacrificed, and their spleens removed aseptically for *in vitro *splenocyte culture. The antigenicity and immunity analysis of the MEAP were performed by EIA. Specific serum IgG antibodies to MEAP were determined with an endpoint EIA using purified MEAP as antigen with 100 μl (2.5 μg/ml) in each well as described previously [13]. The titers were expressed as the reciprocal of highest dilution of sera producing ratio values of 2.1. The selected synthesized B cell epitopes were coated as antigens in 96-well plate with 100 μl (2.5 μg/ml) in each well. The antigenicities of B cell epitopes were detected by EIA with antibody to the MEAP. On the other hand, in order to detect the MEAP antigenicity, the MEAP was coated as an antigen in 96-well plate with 100 μl (2.5 μg/ml) in each well. The MEAP antigenicities against the antibodies to the B, C, D, E, G and I glycoproteins (ABcam) were detected by EIA. All animal work was approved by the Anhui Administrative Committee for Laboratory Animals.

### Virus neutralization tests in vitro

Virus neutralization assay was performed using the procedures described by Cha *et al*. [14] and Kang *et al*. [15]. Neutralization antibodies elicited in immunized mice blood from each group was heated at 56°C for 30 min to inactivate the complement. A 100 μ1 of the heat-inactivated serum was added into a 96-well-flat-bottom microtiter plate for serial dilution (Falcon, Lincoln Park, NJ, USA). A 100 μ1 of 200 × TCID_50 _of the live Sav strain of HSV-2 was added to the plate, sealed and incubated for 18 h at 40°C. Then 100 μ1 of 5 × 10^3 ^viable Vero cells were added to each well and incubated at 37°C for 5 days. The serum dilution factor that neutralized 50% of the virus was determined as the titer. The TCID_50 _was 0.69 pfu as determined by the procedure described by Reed and Muench [16]. Each serum sample was assayed in triplicate.

### Cytokine profiling

The vaccinated mice, from the above, were sacrificed to recover splenocytes 2 weeks after final immunization. The splenocytes were incubated in 96-well flat-bottomed microtiter plates (100 μ1/well of 5 × 10^6 ^cell/ml in RPMI 1640 medium with 10% FCS). The MEAP protein was added at a final concentration of 10 μg/ml. After incubation for 72 h, the splenocyte culture supernatants were collected for cytokine detection. The presence of the cytokines IL-2, IL-4 and IFN-γ in supernatant from the splenocytes cultured from vaccinated mice was examined using commercial murine cytokine EIA kits (Biosource USA), following the manufacturer's instructions.

### Cytotoxic T lymphocyte (CTL) assay

The CTL assay was performed as described earlier [17]. Briefly, mixed splenocytes from 10 mice in each group were stimulated with the MEAP protein (10 μg/ml) in 1 ml of RPMI 1640 medium with 10% FCS in 24-well microplates at 37°C for 6 days. Target cells (NIH 3T3 cells) were prepared by infection with the HSV-2 Sav for 20 h before the assay. The target cells were distributed into triplicate wells of a 96-well plate (5 × 10^3 ^cells per well) and the ratio of effector to target cells was adjusted to 50: 1. The effector and target cells were mixed and incubated at 37°C for 6 h before the supernatant was collected. Lactate dehydrogenase (LDH) activity released into the culture medium was measured with a cytotoxicity assay kit (Promega, USA), according to the manufacturer^' ^s instructions.

### Animal protection experiments

The mice (8 weeks old, BALB/c, female), 10 each group for three groups, were immunized with 4 μg of the MEAP, inactivated vaccine and PBS as control, were challenged with the Sav strain. At 2 weeks after the final vaccination was injected, the anesthetized mice were challenged with the Sav strain. In order to synchronize the estrus cycle at the progesterone-dominated stage, the immunized and sham-immunized mice were subcutaneously injected with 2 mg of progesterone (Jinsite, China) in 50 μ1 of H_2 _O per mouse. At 5 days after the administration of progesterone, the mice of all groups were infected with the Sav strain into vagina and external genital skin. One hour prior to infection, the vaginal closure membrane was ruptured with a saline-moistened cotton swab. The swab was inserted into vagina, twisted back and for five times, then removed and wiped over the external genitalia. To ensure infection, a virus application was repeated 1 h later. The infected mice were examined daily for vaginal inflammation, neurological illness, and death, and then were scored in steps 1-5 depending on the severity of disease as described by the reported papers [17,18]. Vaginal washings were collected on days 1, 2, 3, and 4 post inoculation after the intravaginal challenge with pipetting 100 μl of PBS into the vaginal cavity. The samples were stored at -20°C until used. They were added to 1 ml of the media, and were subsequently infected and titered on Vero cell monolayers by using a plaque assay. Viral neutralization titers in the vaginal secretion samples of the day 4 post inoculation were measured using a micro-neutralization assay described above. The immunized mice were intravaginal challenged with lethal dosages (5 × 10^6 ^pfu: 500LD_50_) of the Sav strain. The vaginal external diseases were examined daily for signs of inflammation and measured mean daily lesion scores on Day 1-14 following infection. The severity of the primary disease was assessed by the lesion scoring system [19,20]. In addition, survival of the vaccinated mice challenged with the Sav strain were daily measured and summarized.

### Statistical analysis

Data were expressed as means ± standard error (SE). Student's *t*-test or analysis of variance (ANOVA) was used for determine the differences among the groups, using the SPSS software package (version 16.0, SPSS Inc., Chicago, IL, USA). A value of *P *< 0.05 was considered statistically significant.

## Results

### Prediction of epitopes independency of MEAP

**Figure 1:**
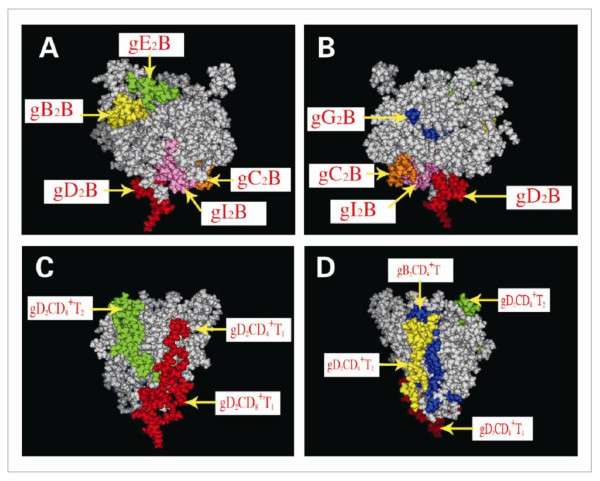
**Three-dimensional of epitopes of MEAP**.1JMA was used as a template for homology modeling of the multi-epitope assembly peptides (MEAPs). The three-dimensional structures of the MEAP were analyzed by software algorithm Moe2008, while the three-dimensional of epitopes were depicted as follows: Three-dimensional B cell epitopes can be seen in A and B, and A is the obverse side opposite to B. Three-dimensional CD4^+ ^T and CD8^+ ^T cell epitopes can be seen in C and D, and C is the obverse side opposite to D.

**Figure 2:**
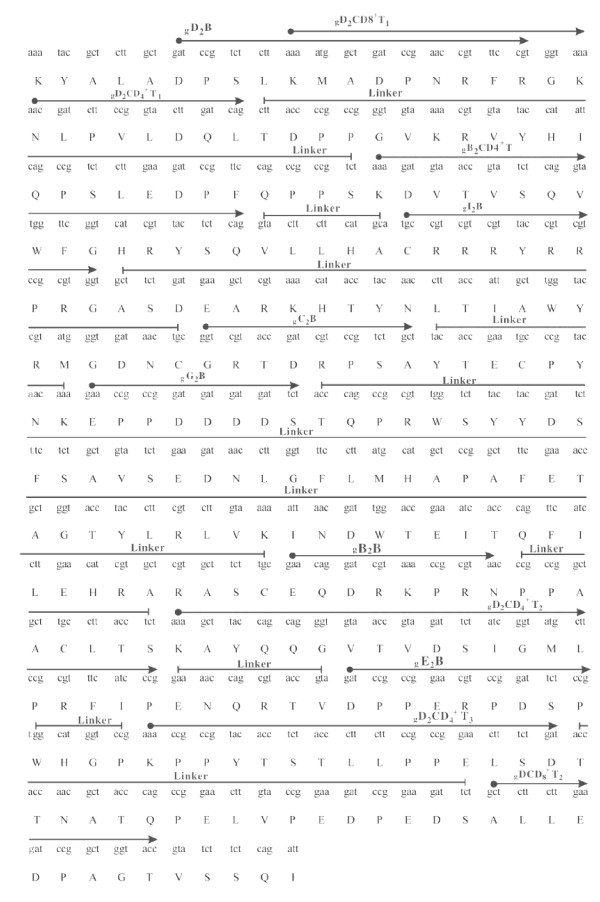
**Design and construction a multi-epitope assembly peptide (MEAP)**. The MEAP were constructed from six B cell epitopes (gB2_466-473_, gC2_216-223_, gD2_6-18_, gE2_483-491_, gG2_572-579 _and gI2_286-295_), four CD4^+ ^T (gD2_21-28_, gD2_205-224_, gD2_245-259 _and gB2_162-177_) and two CD8^+ ^T cell epitopes (gD2_10-20 _and gD2_268-276_) with some non-epitope amino acid sequences of the extracellular fragment (1-290) of HSV-2 glycoprotein D as spacers between epitopes.

The twelve epitopes, including six B, four CD4^+ ^T and two CD8^+ ^T cell epitopes, were used for construction of the MEAPs. The six B (gB2_466-473_, gC2_216-223_, gD2_6-18_, gE2_483-491_, gG2_572-579_and gI2_286-295_), four CD4^+ ^T (gD2_21-28_, gD2_205-224_, gD2_245-259_and gB2_162-177_) and two CD8^+ ^T cell epitopes (gD2_10-20_and gD2_268-276_), were inserted into the extracellular fragment (1-290) of HSV-2 glycoprotein D to construct multi-epitope assembly peptides (MEAPs) by replacing some non-epitope amino acid sequences. Three-dimensional structures of the designed 14 MEAPs were predicted for screening the MEAP in which all the B cell epitopes independently displayed on the surface of the MEAP. The MEAP selected for preparation by genetic engineering was shown in Figure 1 and Figure 2.

### Expression and purification of sumo-MEAP

**Figure 3:**
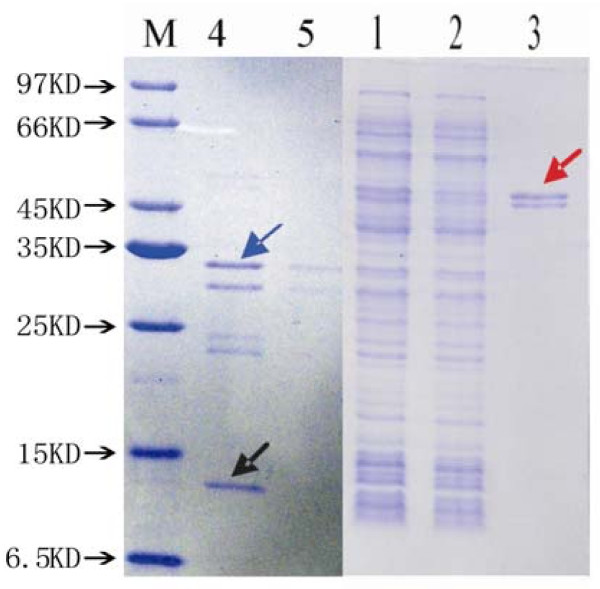
**MEAP purification by Ni Sepharose 6 Fast Flow**. Lane 1: Supernatant. Lane 2: Flow-through. Lane 3: Elution. Lane 4: Sumo-MEAP digested by sumo protease. Lane 5: Purified MEAP. Lane M: Protein marker. Note: Red, blue and black arrow indicate the purified the sumo-MEAP, the MEAP and the sumo tag, respectively.

The gene of the selected MEAP was synthesized chemically and cloned into plasmid pET28a (+)-sumo, the recombinant plasmid pET28a (+)-sumo-MEAP was confirmed by DNA sequencing and was transferred into *E. coli *BL21, the expression of recombinant protein was induced by IPTG and screened by SDS-PAGE analysis. Due to a sumo tag located at the N-terminal of the MEAP, the molecular weight of expressed sumo-MEAP fusion protein was approximately 40 kD. The expressed sumo-MEAP was about 20% of the total bacterial proteins and largely existed in soluble form. The results indicated that the recombinant protein sumo-MEAP was successfully expressed. The sumo-MEAP was purified by Ni Sepharose 6 Fast Flow and detected by SDS-PAGE. The sumo-MEAP was predominantly contained in the elution of 100 mM imidazole. In order to remove the sumo, sumo-MEAP was digested by sumo proteinase at 22°C for 2 h, then purified by Ni Sepharose 6 Fast Flow, the collected eluent was detected by SDS-PAGE (Figure 3).

### Anti-MEAP IgG antibody titers in the sera of immunized mice

**Figure 4:**
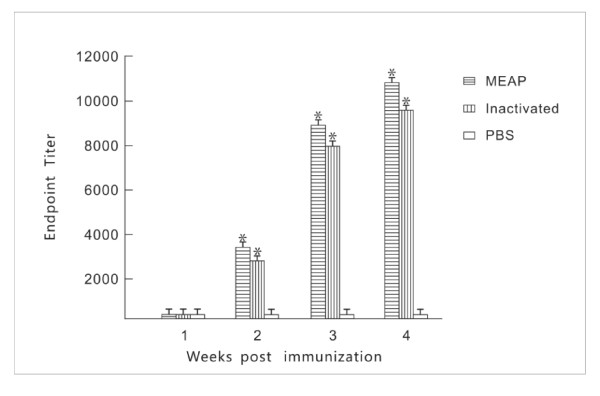
**Antibody response in mice**. Group of BALB/c mice were immunized with the MEAP, inactivated Sav vaccine and PBS. Serum samples (10 mice per group) were collected at various time points, and endpoint antibody titers against HSV-2 were determined by EIA. All the measurements were made in triplicate, with the mean ± standard error (SE) shown. *P *< 0.05 compared with groups immunized with PBS or inactivated vaccine.

All the mice immunized with the MEAP produced the antibodies to the MEAP at the titer from 1: 400 to 1: 10000. The highest titer of antiserum to the MEAP was selected to confirm the immunity of the B cell epitopes by EIA using B cell epitopes as antigens. Also, the MEAP was used as the antigen to detect the corresponding antibodies against gB2, gC2, gE2, gG2, gI2 and gD2 by EIA. All B cell epitopes, except gG2_572-579_, could react with the antiserum against the MEAP, and the MEAP could react with all the antibodies against the six glycoproteins, but only weakly with the antibodies against gG2 (Table 1). The results indicated that the MEAP had good antigenicity and immunity, and implied that the five B cell epitopes, gB2_466-473_, gC2_216-223_, gD2_6-18_, gE2_483-491 _and gI2_286-295_, were displayed on the surface of the MEAP molecule, which was consistent with the prediction. Mice were immunized with the MEAP, inactivated vaccine and PBS by the subcutaneously injection. Serum samples (10 mice per group) were collected at various time points, and endpoint antibody titers against HSV-2 were determined by EIA. The MEAP, inactivated Sav strain and PBS were used to coat in EIA. The result was shown in Figure 4.

**Table 1:** Antigenicities of the MEAP with antibodies to B, C, D, E, G and I glycoprotein of HSV-2 and antigenicities of some B cell epitopes with antibodies to the MEAP

**No**.	Antibodies	EIA OD (450 nm) Value
gD2_6-18_	Ab to MEAP	1.125 ± 0.041
gB2_466-473_	Ab to MEAP	0.977 ± 0.039
gC2_216-223_	Ab to MEAP	0.899 ± 0.031
gE2_483-491_	Ab to MEAP	1.102 ± 0.040
gG2_572-579_	Ab to MEAP	0.182 ± 0.011
gI2_286-295_	Ab to MEAP	0.792 ± 0.029
MEAP	Ab to gD2	1.099 ± 0.037
MEAP	Ab to gB2	0.986 ± 0.031
MEAP	Ab to gC2	0.893 ± 0.029
MEAP	Ab to gE2	1.013 ± 0.041
MEAP	Ab to gG2	0.201 ± 0.011
MEAP	Ab to gI2	0.876 ± 0.031
Control 1	Ab to MEAP	0.116 ± 0.011
Control 2	Ab to gD2	0.121 ± 0.010

The selected synthesized B cell epitopes were coated as antigens. The antibodies to the MEAP reacted with the epitopes. The antigenicities were detected by EIA. The MEAP was coated as an antigen. The antibodies to the B, C, D, E, G and I glycoproteins (ABcam) reacted with the MEAP, and the results can be seen in the Table. No. represents the number of the peptides and the MEAP. Control 1 is a peptide other from the HSV-2 glycoproteins. Control 2 is a antibody other than antibodies to the B, C, D, E, G and I glycoproteins of HSV-2. The O.D. mean values (at 450 nm) are shown in the Table.

### Virus neutralization activity of antisera

Serum samples collected on day 14 were evaluated for their ability to neutralize HSV-2 by serum neutralizing assay. Virus-neutralization activity of the antisera against MEAP, inactivated vaccine and PBS were tested by 50% plaque reduction assay. The neutralizing titer of the antiserum from the mice, which were immunized with the MEAP, had a neutralizing antibody titer of 1/1208. The neutralization titers of antiserum form mice immunized with inactivated vaccine is 1/564. The mice antisera from the PBS-vaccinated mice showed little or no neutralizing activity.

### Th1-type and Th2-type cytokine responses were analyzed in immunized mice

**Figure 5:**
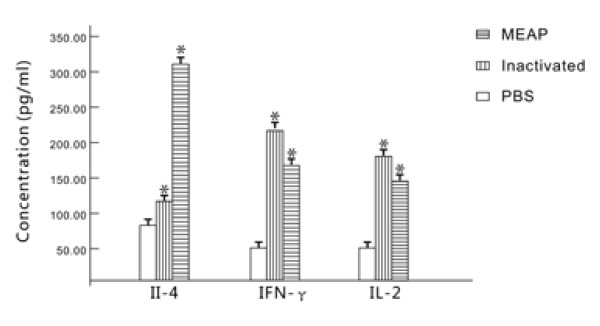
**Cytokine detection in splenocyte culture supernatants from vaccinated mice**. Splenocyte samples (10 mice per group) isolated from the spleens of vaccinated mice on day 14 after the last immunization against the MEAP. After 72 h, the supernatants were collected to examine the levels of the Th1- type cytokine IL-2, IFN-γ and the Th2-type cytokine IL-4 using commercially available murine cytokine EIA kits. All the measurements were made in triplicate, with the mean ± standard error (SE) shown. *P *< 0.05 compared with groups immunized with PBS or inactivated vaccine.

The splenocyte culture supernatant was harvested at 72 h after stimulation to determine the production of different cytokines (IL-2, IL-4 and IFN-γ). Splenocytes from the mice immunized with MEAP induced the highest level of IL-4 (311.23 ± 6.57 pg/ml) among the three groups. The levels of IL-2 and IFN-γ were 149.97 ± 7.53 and 169.11 ± 6.96 pg/ml. This result demonstrated that splenocyte from the mice immunized with MEAP induced predominantly Th-2 (IL-4)-type cellular immune response, because the levels of Th-2 (IL-4)-type cytokines were higher than those of Th1 (IL-2 and IFN-γ)-type cytokines. Splenocyte from the mice immunized with the inactivated vaccine made only small amounts of IL-4 (119.21 ± 6.98 pg/ml); nevertheless, they induced high levels of IFN-γ (219.33 ± 6.99 pg/ml) and IL-2 (181.70 ± 17.43 pg/ml). Splenocyte from the mice that received PBS did not produce any significant cytokine titers (Figure 5).

### Cytokine T lymphocytes (CTL) among splenocyte stimulated with MEAP in vitro

**Figure 6:**
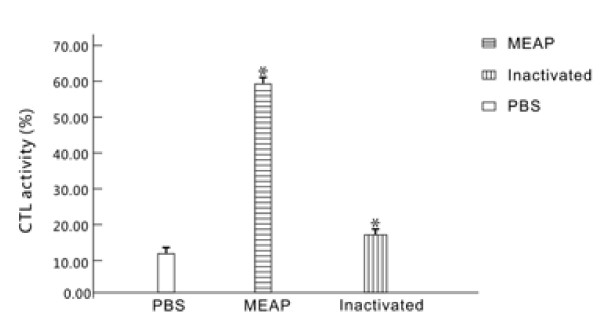
**CTL activity of splenocytes from vaccinated mice 2 weeks after the last immunization**. Splenocyte samples (10 mice per group) isolated from the vaccinated mice were stimulated with the MEAP. After 6 days the CTL activities were detected using a cytotoxicity assay kit by measurement of the LDH released. All the measurements were made in triplicate, with the mean ± standard error (SE) shown. *P *< 0.05 compared with groups immunized with PBS or inactivated vaccine.

The highest level of CTL activity was observed in the group of splenocyte treatment with MEAP in cytotoxicity assay, followed by the groups inoculated with the inactivated vaccine and PBS. The mean CTL activity of MEAP treatment group, inactivated vaccine treatment group and PBS group was 59.94 ± 5.43%, 18.21 ± 1.43% and 12. 21 ± 1.51%, respectively. The CTL responses from the group immunized with MEAP were significantly higher than those from the groups given the inactivated vaccine or PBS (*P *< 0.01) (Figure 6).

### Animal protection experiments

The immunized mice were intravaginal challenged with lethal dosages (5 × 10^6 ^pfu: 500LD_50_) of the Sav strain. The vaginal external diseases were examined daily for signs of inflammation and measured mean daily lesion scores on day 1-14 following infection. The severity of the primary disease was assessed by the lesion scoring system [19,20]. The negative vaccination developed severe diseases and had no effect after 3 days, but the MEAP immunized groups were completely protected from death. The inactivated vaccine immunized mice also showed a high protection level of the lesion score 1 stage from day 4. These results indicated that the immunized mice with the MEAP and the inactivated vaccine had protective effect on HSV-2 infection.

**Figure 7:**
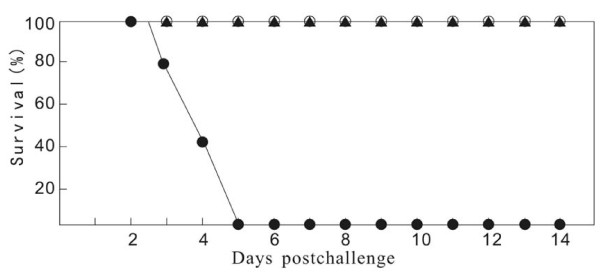
**Survival of vaccinated mice following lethal intravaginal challenge with HSV-2 Sav strain**. Mice were vaccinated three times and then intravaginally challenged with a 5 × 10^6 ^pfu of lethal doses of the strain as described in the section Materials and Methods. Symbols: MEAP- black triangle, inactivated vaccine - white circle, PBS - black circle.

The MEAP and inactivated vaccine immunized groups completely survived during experiments, while the PBS vaccination mice developed severe diseases with all the mice dead after 5 days post the infection (Figure 7).

Virus multiplication was also observed in the genital tracts of all immunized mice on 4th days post infection. The virus was isolated in vaginal tracts with cotton swabs from the immunized mice. Virus titers in the immunized mice on days 1, 2, 3 and 4 post intravaginal infection were significantly reduced than that of the negative control (PBS). Also, each vaccinated group demonstrated that the viral titers decreased with time. On day 1, viral titer of the MEAP immunized mice decreased 4.76 fold than that of the PBS negative control group. Viral titers of the inactivated vaccinated mice were nearly similar to the MEAP immunized mice. On day 4, the virus titer decreased about 4.07 times in the inactivated vaccinated mice and 4.18 times in the MEAP immunized mice than that of the PBS control. These results indicated that virus in MEAP immunized mice and inactivated vaccinated mice were cleared more rapidly from the genital tracts of mice than in PBS treatment mice (*P *< 0.05), and the MEAP has better protective effect against the HSV-2 infection with lethal dosage.

## Discussion

The potential advantages of an epitope based vaccine are as follows. First, epitope based vaccines represent a powerful approach to induce a specific immune response against the selected epitope (s), avoiding the side effects of other unfavorable epitopes in the complete antigen [21]. Second, epitope based vaccine has other considerable advantages, including increased safety, the opportunity to engineer the epitopes rationally for increased potency and breadth, and the ability to focus the immune response on conserved epitopes [22]. Moreover, the epitope based vaccine approach has been shown to be successful in various infectious diseases, such as *Neisseria meningitides *infection, and tuberculosis [23]. In this study, an epitope based vaccine was proposed as a possible candidate vaccine with a remarkable ability to induce protection against HSV-2 infection.

B cell antigenic epitopes from HSV-2 glycoproteins were screened using three software algorithms: Biosun, DNAstar, and Antheprot algorithms. The common results from software were used as candidate epitopes of B cell epitopes. In this study, six B cell epitopes (gB2_466-473_, gC2_216-223_, gD2_6-18_, gE2_483-491_, gG2_572-579 _and gI2_286-295_) were predicted and identified as epitopes (data no shown), which have not been reported. CD4^+ ^T cell epitopes were predicted successively by the APC software algorithm, the lysosome hydrolysis cleavage site software algorithm and the TAP software algorithm. The results of the APC software algorithm provided candidate peptides for the lysosome hydrolysis cleavage site software algorithm. Similarly, the results of the lysosome hydrolysis cleavage site software algorithm provided candidate peptides for the TAP bind software. Some CD4^+ ^T cell immunodominant epitopes, which had higher affinity to TAP than ICP47, were screened as candidate CD4^+ ^T cell epitopes [24]. Four of them (gD2_21-28_, gD2_205-224_, gD2_245-259 _and gB2_162-177_) were identified as epitopes (data no shown), which have not been reported. Here, epitope-prediction program SYFPEITHI, NetMHC and MHCPred were used to analyze the sequence of gD2 according to their different algorithms. Two CD8^+ ^T cell immunodominant epitopes, gD2_10-20 _and gD2_268-276_, were predicted and identified as epitopes (data no shown) [25], which have not been reported.

The gD2, gB2, gC2, gE2, gG2 and gI2 of HSV-2 are largely exposed structural glycoprotein, which have B-cell and T-cell epitopes and are major immunogenic antigen [26]. Ideally, epitope based vaccines should contain both B cell epitopes, which are essential for the protective antibody response, and T cell epitope (CTL epitopes, Th epitopes) that will serve to induce CTL and Th immune responses. Therefore, in the present study, we selected six B cell epitopes (gB2_466-473_, gC2_216-223_, gD2_6-18_, gE2_483-491_, gG2_572-579 _and gI2_286-295_), four CD4^+ ^T cell epitopes (gD2_21-28_, gD2_205-224_, gD2_245-259 _and gB2_162-177_) and two CD8^+ ^T cell epitopes (gD2_10-20 _and gD2_268-276_) from HSV-2 glycoproteins to construct an epitope based vaccine (MEAP). These confirmed epitopes were all inserted into the extracellular fragment (1-290) of HSV-2 glycoprotein D to construct multi-epitope assembly peptides (MEAPs) by replacing some non-epitope amino acid sequences. Three-dimensional structures of the 14 MEAPs were predicted by the software algorithms of Accelrys Discovery Studio and Moe2008 in order to screen a MEAP. All epitopes, especially the B cell epitopes, independently displayed on the surface of the MEAP [12,27,28].

HSV-2 specific IgG response was detected in the sera of mice immunized with MEAP after three boosts, as well as in the mice immunized with inactivated vaccine. This suggests that the MEAP protein and inactivated vaccine were both capable of inducing a significant humoral response in mice. Studies have demonstrated that neutralizing antibodies are important for protection against HSV-2 infection and titers ≥ 1:10 are considered to be indicative of protective immunity [29]. The mean neutralization titer in the mice immunized with MEAP was 1208 after the third boost, and these titers were higher than those induced by the inactivated vaccine (*P *< 0.05). Importantly, these antibody titers were sufficient to protect mice against HSV-2 infection, which indicates the potential of MEAP as a vaccine against HSV-2.

In addition, Cell-mediated immunity plays an important role in efficient protection. The results of cytokine assay and CTL activity indicated that CTL activity and the level of Th2-type cytokines (IL-4) produced in the culture supernatant from mice vaccinated with MEAP was significantly higher than in the groups given inactivated vaccine and PBS. This demonstrated that the multi-epitope vaccine, MEAP, constructed in this study stimulated intensive cellular immunoreaction. Specific CTL are critical in the recovery from infection and the clearance of HSV-2, and the Th2 immune response against HSV-2 is also protective [30], the remarkably increased CTL activity and IL-4 production in the MEAP group may indicated that the MEAP has a higher degree of protection effect.

Challenge experiments showed that the mice that received the MEAP or the inactivated vaccine were completely protected against HSV-2 infection, even though the mean neutralizing antibody titer before challenge of the group vaccinated with the MEAP was lower than that of the group given the inactivated vaccine. This high level of protection, also, is usually related to the MEAP specific neutralizing antibodies [31]. However, we observed high levels of CTL activity in the splenocytes of the mice immunized with the MEAP. This may be contributed to the high levels of protection in the mice immunized with the MEAP, because CTL activity by the T cells was found also to be an essential determinant of protective immunity against HSV-2 [32].

## Conclusions

We have constructed a recombinant multi-epitope assembly peptide (MEAP) from HSV-2 using epitope based vaccine strategies. These results indicated that MEAP was capable of inducing remarkable humoral and cellular immune responses and provided complete protection against lethal challenge in mice. These demonstrate that the MEAP may be a promising subunit vaccine candidate for the prevention of HSV-2 infection. The present study also provided useful information for the further development of multi-epitope vaccine for human.

## Competing interests

The authors declare that they have no competing interests.

## Authors' contributions

XW responsible for conducting experiment and writing the manuscript. GX responsible for conducting some of experiment and writing some of the manuscript. JL was responsible for epitopes prediction and tested antibodies activity *in vitro *, and DY was responsible for cytokine profiling. WG was responsible for cytotoxic T lymphocyte assay. MP responsible for animal protection experiments, YL and JL responsible for conducting experimental design and conducting experiment. All authors read and approved the final manuscript.
